# Chicago Veterinary Society

**Published:** 1897-07

**Authors:** Lawrence Campbell

**Affiliations:** Secretary


### CHICAGO VETERINARY SOCIETY.

MEETING called to order by the President, June 10, 1897, the first order of business being a banquet, at which twenty-two members were present. After the banquet the minutes of the previous meeting were read and approved. Twenty-one letters were then read by the Secretary from veterinarians throughout the State, many of whom were assistant State veterinarians, and who wrote notifying the Society that they had resigned their office owing to the appointment by the Governor of an unqualified man to the office of State veterinarian. Moved by Dr. Robertson, seconded by Dr. E. L. Quitman, that the Secretary send to the *Breeders' Gazette* a synopsis of these minutes with the names of the assistant State veterinarians resigned, for publication, and that a copy of the paper be mailed to each veterinarian in the State; carried.

The next order of business was the reading of papers. Dr. Henderson was called on for his paper, "Meat- and Milk-inspection in Chicago, and the Efficiency of the Present Laymen Inspectors." The Doctor presented extracts from the present unenforced laws, and also stated the stringent laws now in force in Minneapolis. The article was a most excellent one, and complete in all its departments.

Dr. Robertson presented a paper relating to the law governing veterinary work in the police department. This also was an excellent statement of present facts.

Dr. P. Quitman was unable to make any report in regard to the connection of a veterinarian with the fire department. Quite a long debate was held by the members as to drafting a bill for passage by the city council to place veterinarians as assistants in the city health department. Moved by Dr. Fisk, seconded by Dr. Campbell, that the Legislative Committee call on the Corporation Council or elsewhere and ascertain what are the chances to create a veterinary department in connection with the health department; carried.

Moved and seconded to close discussion; carried. On application and examination by the Board of Censors, Dr. H. H. McEvers was admitted to membership.

The hour being midnight, it was moved and seconded to adjourn.

LAWRENCE CAMPBELL,
Secretary.

